# Reports on the Hospitals of the United Kingdom

**Published:** 1910-05-28

**Authors:** Henry Burdett


### REPORTS
### ON THE
### Hospitals of the United Kingdom.

By SIR HENRY BURDETT, K.C.B., K.C.V.O.

### XLVIII.—THE ROYAL ALBERT EDWARD INFIRMARY, WIGAN.

This hospital was established in 1874; the Gidlow wing was built in 1884, the Johnson children's wing in 1890, the new mortuary in 1894, the Gidlow Memorial wing and nurses' dining-hall in 1895, the isolation block in 1900. It contains 156 beds, the average daily number occupied being 134. This hospital commands the sympathy and support of the working-classes. The workpeople's contributions in 1908-09 amounted to £8,363, or upwards of two-thirds of the total income. The practical interest and organisation which make this hospital remarkable include 27 horse and 12 hand ambulances, which are stationed at various large works throughout the district; each of the works bears the cost of its own ambulance. We congratulate the men of Wigan upon their foresight and prudence in maintaining these ambulances, and commend their action to other large working districts all over the country. We know of no hospital but this at which upwards of two-thirds of the total annual revenue is supplied by the workmen who use the hospital.

The plan on which this hospital is built, though a good one, cannot be compared with a later effort, the Halifax Infirmary plan, by the same architects, Messrs. Worthington and Son. We noticed one detail which it may be useful to mention—namely, that the encaustic tiles in the main corridors are very loose, a fact which indicates that they are unsuitable for the hard wear of a hospital unless they are laid at the commencement with special care, and are subsequently maintained, with the aid of weekly tests, in the most perfect order.

### Its Present Condition.

We are glad to be able to testify that the Wigan Infirmary is well organised and a smoothly working hospital. It was a pleasure to inspect it, and although Miss K. V. Macintyre, the matron, was away on holiday the most careful inspection of the details of each department proved that the whole institution is well kept and all the sisters are most efficient. This says much for the management, seeing that most of the sisters have been trained at Wigan. We regretted Miss Macintyre's absence, because we should have liked to have congratulated her upon the atmosphere and efficiency which dominated the whole of her department. We found the assistant matron, Miss P. E. Fletcher, most helpful, knowledgeable, and ready to help in every possible way. Dr. Readman, who accompanied us during our inspection, was enthusiastic in his testimony to the value of the infirmary, to the interests of which he is sincerely attached.

We are able to say of this hospital that the floors and fittings, and all the appurtenances of the wards and departments, are good throughout. The attention to detail which was apparent everywhere was very cheering, and was well exhibited in the special and attractive brightness of the numerous copper sinks and vessels which form rather a feature of this hospital. The same may be said of the stores and linen rooms, all of which are good, the latter having ample space and a really splendid table, an essential which is sadly missing in many hospitals we have inspected. The stores were well selected, the tea and bread being specially good. The supply of meat here, however, as at some of the other hospitals and Poor Law Infirmaries in this district, constitutes a difficulty, owing to the failure of the contractors to fulfil their contract with invariable efficiency. All the wards are bright, attractive, and not over-crowded. The children's wards were specially noteworthy, for here the children are well and tenderly cared for. Indeed, these wards presented a striking contrast to some we have inspected elsewhere. Another feature of the hospital which is noteworthy is the fact that the kitchen is under a lady cook. We found this department well kept, and were struck by the undoubted fact that all the supplies were well cooked and appetisingly served. Altogether we should say that it is a good thing when ill to be a patient at the Wigan Infirmary.

### Pressing Needs.

At this hospital operations usually take place only on fixed days in each week, and it would appear that the number of hours during which the operation theatre is sometimes in use are too prolonged, if due consideration is to be given to the resident medical and nursing staffs. It is apt to be forgotten that the sister and nurses and the resident medical officers, when one of the latter has to give all the anæsthetics, have to remain in the theatre from the beginning to the end of each day's work. Sometimes the temperature is maintained at 80°, and the strain upon the health and energies of these officers is often excessive. At Wigan, in addition to the usual operations the practice prevails for one of the staff to operate always in the evening. We would venture to suggest that if this plan be unavoidable, the days on which there are evening operations should be other than those set apart for day operations. The hospital has great need of a new casualty department with a small theatre attached, and an ample recovery room. With this accommodation it should be practicable to lessen considerably the pressure on the present theatre staff. A new out-patient department is also a desirable feature, and better accommodation for the staff should be provided. With these additions and changes, having regard to the atmosphere and air of Wigan, and the attractive character of many of its houses and buildings, with its wide streets and open spaces, the Wigan Infirmary should be rendered one of the most popular hospitals in the North of England. We are glad to have met the General Superintendent and Secretary, Mr. William Taberner, who is enthusiastically interested in his work, and fully conscious of the needs and requirements of the hospital.

---

**Radcliffe Infirmary, Oxford,** will be reported on next week.

### The Hospital World.

